# Hyaluronic Acid-Based Nanomaterials as a New Approach to the Treatment and Prevention of Bacterial Infections

**DOI:** 10.3389/fbioe.2022.913912

**Published:** 2022-06-08

**Authors:** Reza Alipoor, Mohammad Ayan, Michael R Hamblin, Reza Ranjbar, Somaye Rashki

**Affiliations:** ^1^ Student Research Committee, Hormozgan University of Medical Sciences, Bandar Abbas, Iran; ^2^ Sina Higher Education Institute, Kashan, Iran; ^3^ Laser Research Centre, Faculty of Health Science, University of Johannesburg, Doornfontein, South Africa; ^4^ Molecular Biology Research Center, Systems Biology and Poisonings Institute, Baqiyatallah University of Medical Sciences, Tehran, Iran; ^5^ Student Research Committee, Kashan University of Medical Sciences, Kashan, Iran; ^6^ Department of Microbiology and Immunology, Faculty of Medicine, Kashan University of Medical Sciences, Kashan, Iran

**Keywords:** hyaluronic acid, nanoparticles, antimicrobial agents, antibacterial coatings, controlled release

## Abstract

Bacterial contamination of medical devices is a great concern for public health and an increasing risk for hospital-acquired infections. The ongoing increase in antibiotic-resistant bacterial strains highlights the urgent need to find new effective alternatives to antibiotics. Hyaluronic acid (HA) is a valuable polymer in biomedical applications, partly due to its bactericidal effects on different platforms such as contact lenses, cleaning solutions, wound dressings, cosmetic formulations, etc. Because the pure form of HA is rapidly hydrolyzed, nanotechnology-based approaches have been investigated to improve its clinical utility. Moreover, a combination of HA with other bactericidal molecules could improve the antibacterial effects on drug-resistant bacterial strains, and improve the management of hard-to-heal wound infections. This review summarizes the structure, production, and properties of HA, and its various platforms as a carrier in drug delivery. Herein, we discuss recent works on numerous types of HA-based nanoparticles to overcome the limitations of traditional antibiotics in the treatment of bacterial infections. Advances in the fabrication of controlled release of antimicrobial agents from HA-based nanosystems can allow the complete eradication of pathogenic microorganisms.

## Introduction

**FIGURE 1:**
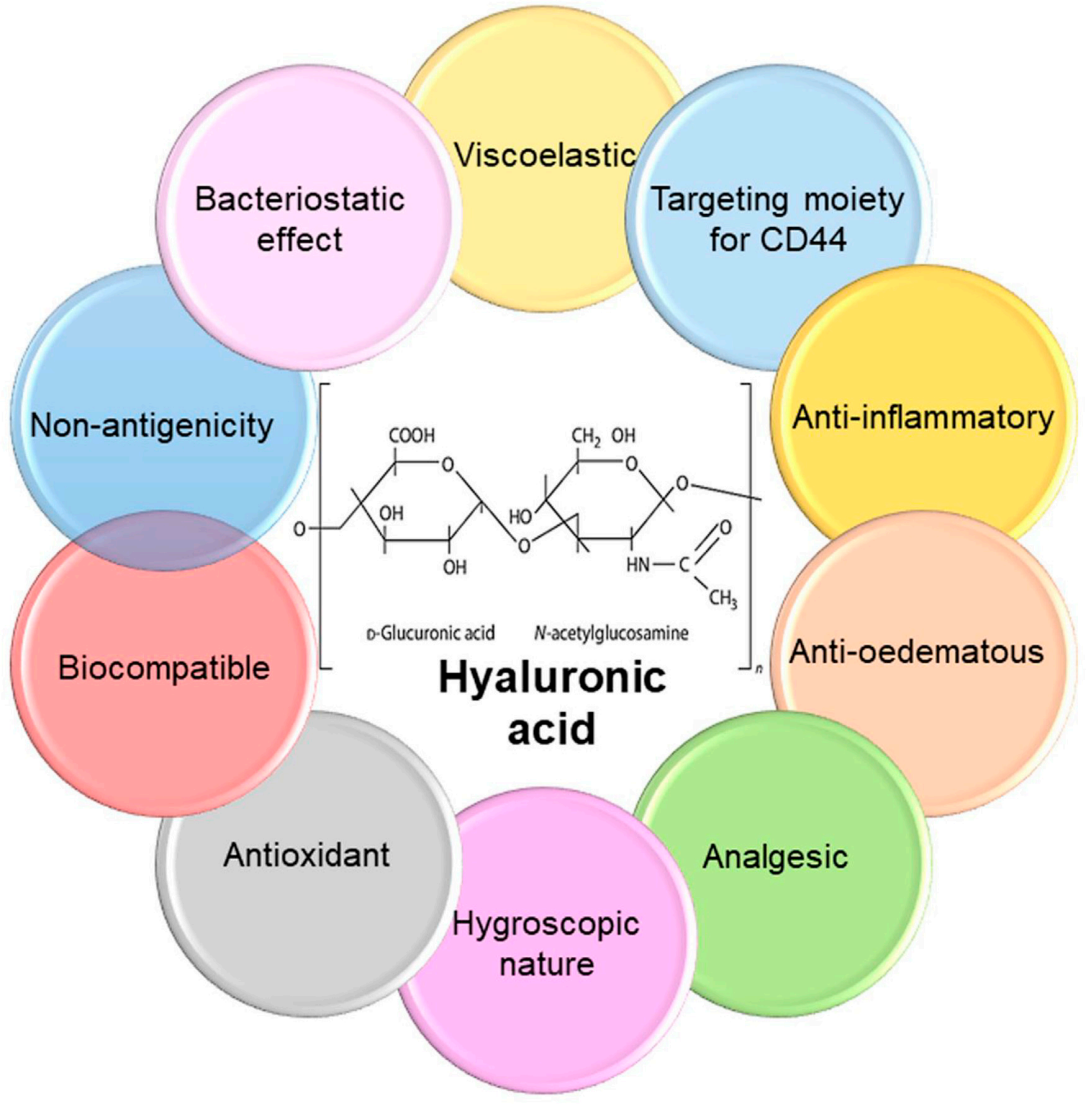
Chemical structure and properties of hyaluronic acid. HA is a natural biodegradable linear polyanionic macromolecular mucopolysaccharide comprised of two repeating saccharide units of glucuronic acid and N-acetylglucosamine linked alternatively by β-1,3- and β-1,4-glycosidic bonds. HA has numerous attractive features that make it suitable option for biomedical applications.

The microbial contamination of medical equipment caused by bacterial or fungal pathogens is an important factor in the transmission of nosocomial infections (Francolini et al., 2015; Abed et al., 2022; Panahi et al., 2011; Rashki et al., 2021a; Marzhoseyni et al., 2022; Mirzaei et al., 2020). Moreover, the alarming rise in antimicrobial-resistant species caused by the overuse of antibiotics now poses serious health and economic threat worldwide (Marcello et al., 2021). Based, finding new materials with proper antibacterial landscapes as alternatives to antibiotics to combat bacterial infections has opened a novel field of research (Abed et al., 2022; Marcello et al., 2021; Yazdanian et al., 2022; Lashkari and Ranjbar, 2021; Boroumand et al., 2021; Rashki et al., 2021b; Khan et al., 2021). In this regard, cationic polymeric materials have attracted much interest, because of their intrinsic bactericidal activity and useful antibacterial effects (Khoobi et al., 2020). Cationic polymers comprise a group of environmentally-friendly antimicrobial agents, which can affect bacterial cell membranes without inducing microbial resistance, regardless of existing antibiotic resistance. Glycosaminoglycan (GAG) was first isolated by Karl Meyer and John Palmer (1934) from bovine vitreous tissue and called hyaluronic acid (HA); in Greek hyaloid means vitreous plus uronic acid (Gupta et al., 2019). HA is an important component of the extracellular matrix (ECM), found in bone marrow, synovial fluid, and the articular cartilage of mammals (Wang et al., 2019). HA has been described as “nature’s moisturizer” due to its hydrophilic (water-loving) nature. Microbial fermentation can be used as a source of HA, by culturing *Escherichia coli, Streptococcus zooepidemicus, Bacillus subtilis*, etc. (Gupta et al., 2019). HA polymer has attractive properties, including biocompatibility, biodegradability, viscoelasticity, non-inflammatory, non-immunogenic, and low toxicity (Figure 1). Hence, HA has been widely applied in numerous drug delivery systems, such as nanoparticles (NPs), microspheres, polyelectrolyte microcapsules, gels, films, cationic polymer genes, liposomes, nano-emulsions, and so on (Oh et al., 2010; Almalik et al., 2013; Liu et al., 2018). It also serves as a carrier for a variety of drug delivery systems such as receptor-mediated drug targeting in cancer treatment, delivery of peptide, protein, and nucleotide therapeutics, and imaging agents because of its strangeness in detecting receptors whose expression has increased in different diseased cells (Oh et al., 2010; Kim et al., 2018). In the medical industry, HA is used to stabilize cartilage matrix, as a wound dressing for diabetic foot ulcers (DFUs), as an ophthalmic biomaterial in eye surgery such as corneal transplantation, retinal detachment repair, and cataract surgery, and as viscosupplement injections into the knee to increase the synovial fluid viscosity, which cushions and lubricates the joint (Kogan et al., 2007; Huang and Huang, 2018; Prajapati and Maheriya, 2019). In the field of drug delivery, HA has gained popularity due to its advantages such as biodegradability; biocompatibility; ease of chemical modification; high potential drug loading; and intrinsic targeting properties due to selective interactions with receptors like cluster determinant 44 (CD44), hyaluronan receptor for endocytosis, and tool-like receptors (TLRs) (Prajapati and Maheriya, 2019). Low-molecular HA is utilized in cosmetics as a highly effective humectant, antioxidant, and stimulating agent for collagen formation; cell proliferation, reconstruction of soft tissue, and cytotoxic are thought to be important factors in battling the aging process (Kogan et al., 2007; Schmidt and Leach, 2014). Therefore, with the increase in board application platform of HA and due to its useful biological properties, HA can be easily manipulated to optimize its physical and biological features for a wide range of applications in the biomedical, pharmaceutical, food, and cosmetic industries.

Here, we summarize the structure, properties, and production of hyaluronic acid. Moreover, this review highlights recent works on nanostructured HA in the treatment of bacterial infections. Finally, the application of various HA platforms as a carrier in drug delivery will be discussed.

## Hyaluronic Acid Properties and Antibacterial Activity

HA, also known as hyaluronan, is a linear polymer, water-soluble, viscoelastic containing disaccharide repeats of (Francolini et al., 2015; Rashki et al., 2021a)-glucuronic acid (GlcUA)- (Francolini et al., 2015; Panahi et al., 2011)-*N*-acetylglucosamine (GlcNAc) (Figure 1) (Boeriu et al., 2013). Hyaluronan belongs to the GAG family, containing keratin sulfate, heparin/heparan sulfate, and chondroitin/dermatan sulfate. Structurally, GAGs contain repeating disaccharides of a sulfated (or carboxylated) amino sugar (glucosamine or galactosamine) and a hexose (galactose, iduronic acid, or glucuronic acid) forming a long unbranched polysaccharide chain (DeAngelis, 2012). HA is the only member of the GAG family which is not sulfated.

Physiologically, HA has well-documented roles in organs and body fluids. Generally, HA can affect various cellular processes (differentiation, proliferation, development, and molecular recognition), and also physiological processes (lubrication, hydration balance, matrix construction, and steric interactions). In clinical settings, HA has been used for the management of various medical conditions, such as ophthalmological surgery (Aragona et al., 2002), arthritis (Moreland, 2003), tissue volume augmentation (Klein, 2011), and wound healing (Kawano et al., 2021), due to its aforementioned physicochemical properties (Jin et al., 2010). The molecular weight of hyaluronan is the main factor that governs its biological effects. In this regard, high molecular weight hyaluronan (>5 × 10^5^ Da) can act as a tissue filler, an immunosuppressant, and an antiangiogenic biopolymer. Medium molecular weight (2 × 10^4^–10^5^ Da) plays a role in ovulation, embryogenesis, and wound healing. Low molecular weight oligomers with 15–50 repeating disaccharide units (6 × 10^3^–10^4^ Da) have immunostimulatory, inflammatory, and angiogenic effects. Finally, short hyaluronan chains (400–4,000 Da) have anti-apoptotic effects and can induce heat shock proteins (Stern et al., 2006).

The preparation of small oligosaccharides and low molecular weight hyaluronan can be achieved by controlled hydrolysis of macromolecular hyaluronan, using physical degradation (high temperature and pressure), irradiation (γ- ray, microwave, or electron beam), metal-catalyzed radical oxidation, acidic depolymerization, ozonolysis, or treatment with hyaluronidase enzyme (Boeriu et al., 2013). The molecular weight and concentration of HA govern its anti-microbial properties (Romanò et al., 2017). Besides, the antibacterial effect of intravesicular HA has been demonstrated in chronic UTI, where it was proposed to replace the loss of the natural GAG layer coating the bladder wall (Damiano and Cicione, 2011). Also, HA has dose-dependent bacteriostatic activity against several planktonic microorganisms (Pirnazar et al., 1999). Radaeva et al. found that HA showed inhibitory activity against some *Pseudomonas* species (Radaeva et al., 2001), and Ardizzoni et al. (2011) measured the inhibitory activity of HA against fifteen standard microbial species (ATCC), covering clinically relevant fungal and bacterial strains. Despite no inhibitory effects of HA against *C. albicans* or *E. coli* ATCC 13768, HA did exert dose-dependent growth inhibition against *Streptococcus mutans*, Enterococci, Staphylococci, two *P. aeruginosa* strains, *E. coli* strains, *C. parapsilosis,* and *C. glabrata*, while *S. sanguinis* responded only to the highest HA dose (4 mg/ml) (Ardizzoni et al., 2011). Additionally, HA can decrease bacterial adhesion and subsequent biofilm formation. The HA antibacterial efficacy may be limited because of mainly acts as a passive protective barrier. For example, the type and loading of bacteria, the local environment, etc. can affect the antiadhesive and antibiofilm activity of HA. On the other hand, the HA protective effects may be counteracted by bacterial secreted hyaluronidase, an enzyme that breaks down HA (Hynes and Walton, 2000). Different clinical settings have provided evidence for the antibiofilm effects of HA and its derivatives, as a non-antibiotic platform with a good safety profile, high biocompatibility, and anti-adhesive properties. Given the rapid hydrolysis of HA by natural hyaluronidase enzymes, the use of pure HA would not be appropriate as an antimicrobial coating. Because of its strong hydrophilicity, a HA-based hydrogel used alone would also not be a suitable coating with sufficient mechanical stability in a water-based environment such as the human body (Gaetano et al., 2018). Therefore, to overcome these drawbacks and increase the biological half-life of HA, chemically crosslinked HA nanogels were created because they are often more stable than the physically crosslinked analogs. Crosslinked HA nanogels require both HA molecules and crosslinkers to be spatially confined within nano-sized compartments (Ossipov, 2010). Following the process, the HA hydrogels retain the biocompatibility and biodegradability of the unaltered material. HA crosslinking can be accomplished in two ways: by directly adding a cross-linker and generating the three-dimensional (3D) network, or by pre-modifying the HA chains with functional groups that are likely to be crosslinked. The latter results in the formation of active moieties, which add new functionalities to the hydrogel. The carboxyl group, hydroxyl group, and N-acetyl group (mainly carboxyl group) of HA disaccharide units are three sites that may undergo chemical modification (Pérez et al., 2021). One alternative could be an antibacterial HA-based composite scenario, which needs further study and testing in a large-scale clinical trial according to regulatory requirements.

## Production of Hyaluronic Acid

HA can be produced on an industrial scale, either by being obtained from animal organs or else by fermentation of genetically modified bacteria in large-scale culture vessels. These methods have been extensively used to produce HA molecules (Mw > 1 MDa) for cosmetic and biomedical uses. These types of HA have a longer biological half-life while retaining their physiological activity (Liu et al., 2011).

### Extraction From Animal Tissues

Since the first isolation of HA in the early 1930s, this molecule has been extensively isolated from various tissues of animals, such as rooster comb, bovine synovial fluid, and human umbilical cord (Meyer and Palmer, 1934; Liu et al., 2011). Animal-derived HAs generally have high molecular weights. In particular, the rooster comb is a valuable source of HA due to its high concentration (7,500 μg HA/g of tissue) (Fraser et al., 1997). Despite the solubility of HA in water, obtaining extremely pure high molecular weight HA from animals is difficult due to the formation of complexes between HA and other biomacromolecules, such as proteoglycans. Different techniques have been proposed to separate HA from these complexes, including HA ion-pair precipitation (e.g., with cetylpyridinium chloride), protease digestion (e.g., trypsin, papain, pronase, or pepsin), HA non-solvent precipitation, organic solvent precipitation, detergents, etc. (Boeriu et al., 2013). Despite all the efforts to purify HA, contamination of animal HA with nucleic acids or proteins remains problematic. The amount and type of impurities depend on the precise animal source. In this context, HA obtained from the human umbilical cord or bovine vitreous body contains higher amounts of nucleic acids and proteins, than HA isolated from bacterial origin or rooster comb (Shiedlin et al., 2004). Commercial animal-extracted HA has a molecular weight between several 100,000 and 2.5 million Da (Kogan et al., 2007).

### Bacterial Production

**FIGURE 2:**
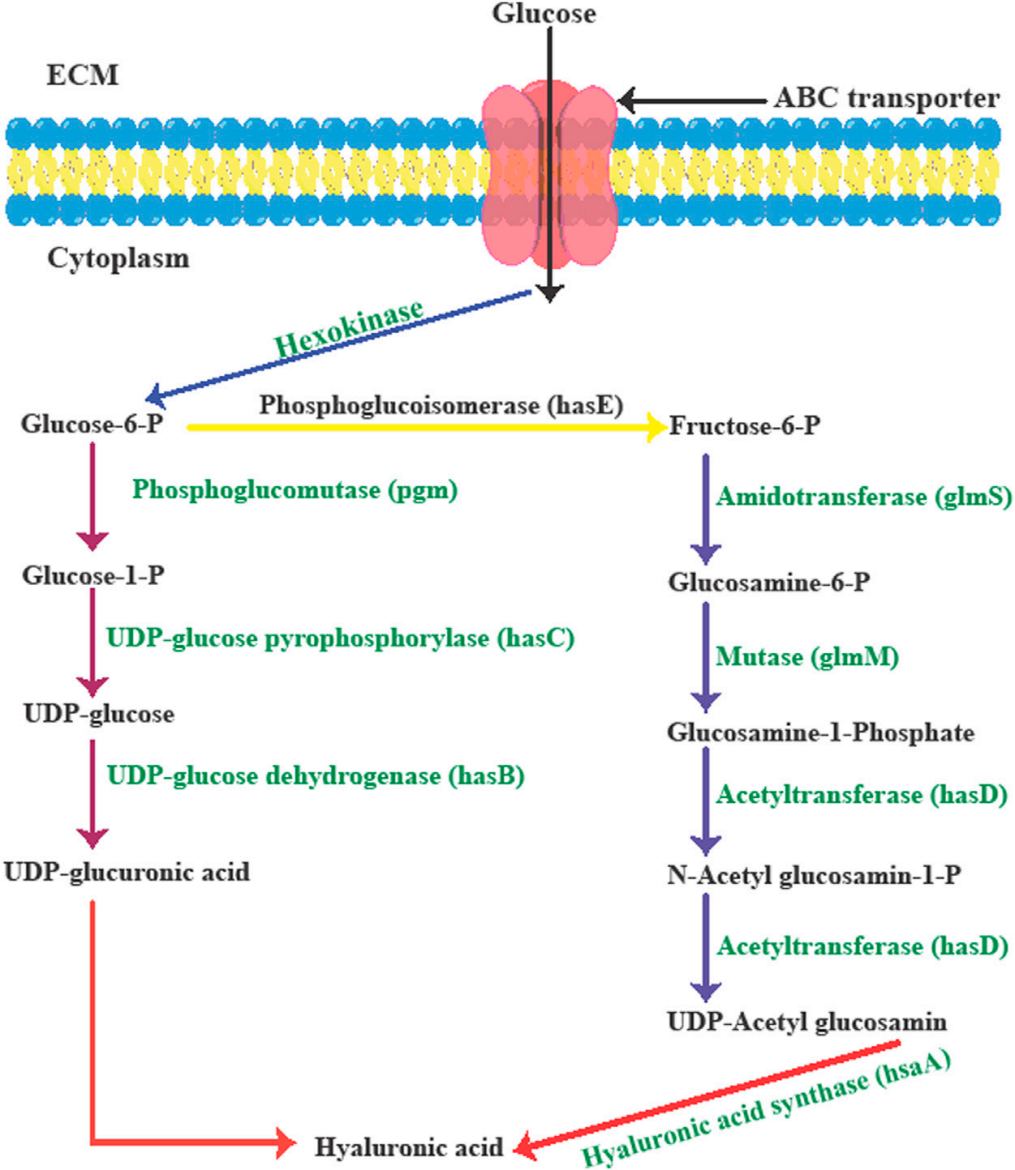
The biological synthesis pathway of hyaluronic acid in *Streptococcus zooepidemicus*. Adapted from Ref (Sze et al., 2016b) with changes.

Among bacterial-based HA production methods, group C streptococci (non-pathogenic in humans) are commonly used to produce HA, because of better hyaluronan synthesis compared to *P. multocida* (an animal bacterial pathogen), or group A streptococci. In this regard, the main group C *streptococcus* strains is *S. equi* subsp. Zooepidemicus and *S. equi* subsp. Equi (Izawa et al., 2009). The HA biosynthesis pathways in *S. zooepidemicus* can be classified into two distinct routes to form HA precursors (Figure 2).

Streptococci strains use glucose to produce the sugar backbone of HA *via* the hyaluronic acid synthesis operon (hasA, hasB, hasC, hasD, and hasE). During the first set of reactions, glucose-1-phosphate is produced from glucose-6-phosphate *via* phosphoglucomutase (pgm) activity. To produce uridine diphosphate (UDP)-glucose, UDP-glucose pyrophosphorylase (hasC) transfers the uridine triphosphate (UTP) phosphate group to glucose-1-phosphate. In the final step, UDP-glucose dehydrogenase (hasB) oxidizes the primary alcohol of UDP-glucose to produce UDP-glucuronic acid (UDPGlcUA, the first HA precursor). During the second set of reactions, phosphoglucoisomerase (hasE) can form fructose-6-phosphate from glucose-6-phosphate, which is then tagged with an amido group from a glutamine residue through amidotransferase (glmS), leading to glucosamine-6-phosphate. Next, mutase (glmM) rearranges the phosphate group of glucosamine-6-phosphate to form glucosamine-1-phosphate. In the next step acetylated and phosphorylated forms of glucosamine-1-phosphate are produced by an acetyltransferase and pyrophosphorylase (hasD) to produce UDP-N-acetylglucosamine (UDP-GlcNAc) as the second HA precursor. Next, hyaluronic acid/hyaluronan synthase (hasA) combines the two precursors to form the HA polymer. Because multiple intermediates are utilized in the biosynthesis of HA in the bacterial cell wall and this consumes significant amounts of energy, the formation of biomass and the generation of lactate depends on glycolysis (Chong et al., 2005; Sze et al., 2016a).

HA products derived from pathogenic streptococcal strains have been associated with some safety concerns. To overcome this obstacle, safe microorganisms have been genetically modified by the expression of hyaluronan synthase enzymes from either *P. multocida* or *streptococci*. Host bacterial strains such as *E. coli*, *Enterococcus faecalis*, *Agrobacterium sp*, *Bacillus subtilis*, and *L. lactis* have been genetically engineered for efficient HA production (DeAngelis et al., 1993; Yu and Stephanopoulos, 2008; Mao et al., 2009; Widner et al., 2005; Mao and Chen, 2007; Prasad et al., 2010; Chien and Lee, 2007; Prasad et al., 2012). Recently, Gram-positive bacteria (*B. subtilis*) as well as Group *A and C Streptococci* have become well-known industrial producers of different compounds such as HA (Novozyme). Besides Gram-positive bacteria, several Gram-negative bacteria (e.g., *E. coli*) do not express an important enzyme in the HA synthesis machinery, or else members of the pathway are only found at very low levels. This makes most strains of *E. coli*, such as JM109, unsuitable for HA synthesis. Nevertheless, the expression of HAS (pmHAS) from *P. multocida* and UDP-glucose dehydrogenase (kfiD) from *E. coli* K5 could allow the production of HA in different *E. coli* strains (e.g., JM109). In addition, the addition of glucose and glucosamine (precursors of HA) into the culture media in this approach can increase the efficiency of HA production (yield of 3.8 g/L and molecular weight of 1.5 MDa) (Figure 5) (Sze et al., 2016a).

### Cell-Free (*In Vitro*) Generation of Hyaluronic Acid

While bacterial expression methods can manufacture HA in small scale fermenters, HA synthesis on a larger scale increases the viscosity of the media resulting in non-uniform size products, poor mixing, and a low oxygen mass transfer rate, thus making yields more than 6–7 g/L difficult to attain (Sze et al., 2016b).

To produce monodisperse HA, the parameters of bacterial fermentation for HA synthesis must be known and controlled to achieve this objective. Moreover, the use of animal-derived HA products also faces concerns due to stimulation of immunity mediated by bacterial cell-based contamination (HA binding proteins, nucleic acids, toxins, etc.,). Hence, the use of a cell-free HA production system (*in vitro*) has been proposed as an alternative to overcome these obstacles. In this system, Class I HA synthase enzymes cannot be used because they are integral membrane proteins. These HA synthases are often studied in the field of membrane fractionation, and would not be suitable for large-scale production. The immobilization of enzymes in the cell wall of yeast cells could be considered an alternative to produce various human oligosaccharides. For example, glycosyltransferases can be localized in the yeast cell wall glucan where they retain their enzymatic activity (Shimma et al., 2006). *P*. *multocida* Class II HA synthase has been reported to be a more promising enzyme for *in vitro* production of HA. This enzyme is not an integral membrane protein, and its recombinant modified structure (truncation of 216 carboxy terminal amino acids) retains HA synthase activity. It showed cytoplasmic localization after expression in *E*. *coli* (Jing and DeAngelis, 2000). Moreover, *P*. *multocida* HA synthase carries out two distinct transferase reactions, one of which could be disabled by altering the DXD motif amino acids at the active site, while the activity of the other transferase would be retained.

## Hyaluronic Acid as Carrier in Drug Delivery Systems

HA possesses several groups suitable for chemical modification, such as carboxyl, hydroxyl, and *N*-acetyl groups (Huang and Huang, 2018). Since HA is a biodegradable, biocompatible, and highly efficient cargo-loading polymer with easily chemical modifications, it has become an attractive carrier in the drug delivery field. Consequently, HA and HA-based composites have been widely investigated in several drug delivery platforms, such as hydrogels, nanoparticles (NPs), nanoemulsions, polyelectrolyte microcapsules, and microspheres, as well as cationic polymer film delivery systems.

### Protein-Hyaluronic Acid Conjugates

**FIGURE 3:**
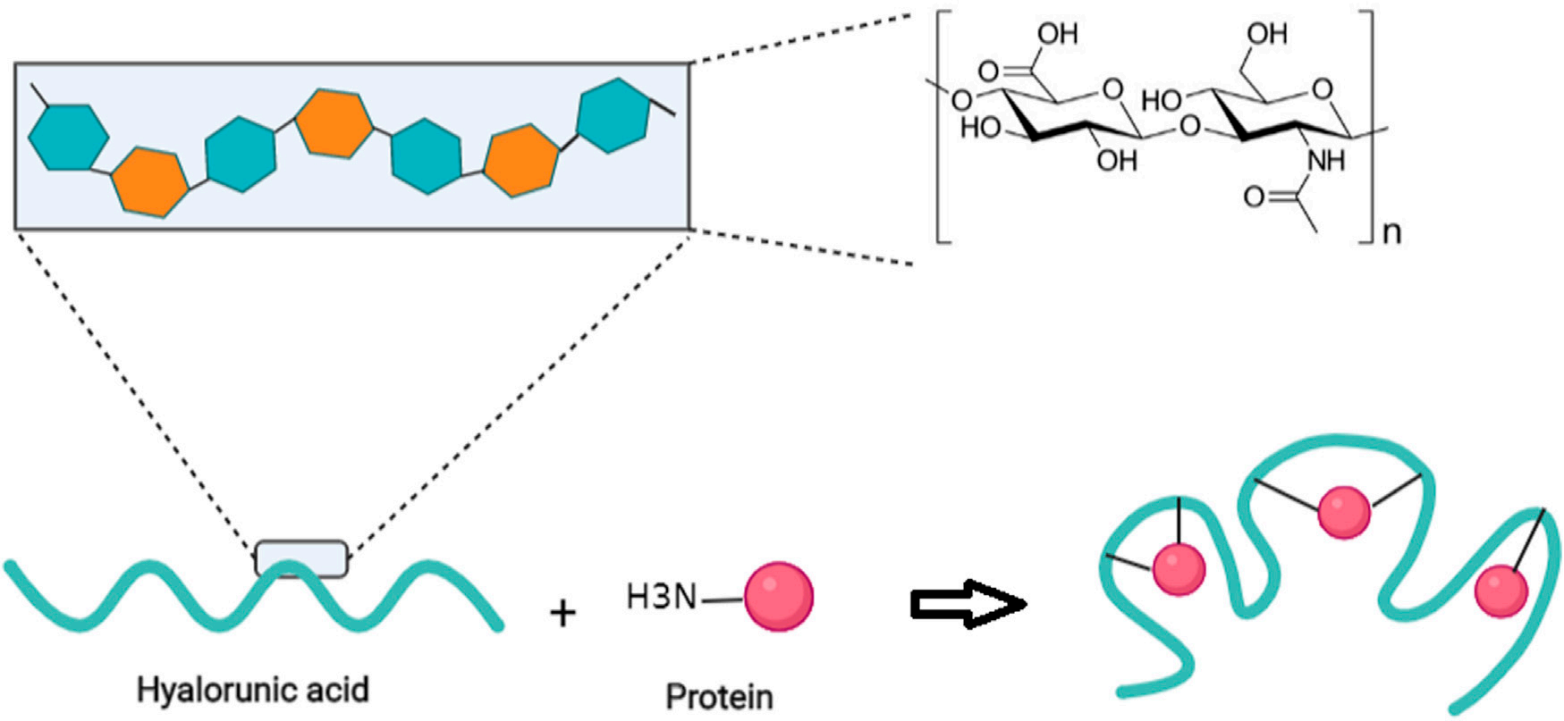
A schematic illustration of the random conjugation of free amino groups of proteins to carboxylic groups of HA polymer. Adapted from refs (Mero and Campisi, 2014) (61) (Ghosh et al., 2017) (59) (Jing and DeAngelis, 2000) (57) (Prasad et al., 2012) (56) (Chien and Lee, 2007) (54) (Mao and Chen, 2007) (52) (Mao et al., 2009) (51) (Mao et al., 2009) (44) (Shiedlin et al., 2004) (45) (Pérez et al., 2021).

Duncan *et al.* proposed that HA could be used as a polymer for the conjugation to proteins in a polymer-masking-unmasking protein therapy (PUMPT) approach (Hardwicke et al., 2008). Although biomacromolecules, like peptides and proteins, have some potential to manage ocular diseases as a novel approach (Ghosh et al., 2017), delivery of these bioactive molecules still encounters many obstacles, including degradation, and a short half-life *in vivo* (Ibeanu et al., 2020)*.* HA is a bioresponsive polymer which can bind to ribonuclease A (RNase A) and trypsin in the PUMPT concept. These model proteins were modified so that amino groups of proteins could be coupled to the carboxylic groups of HA polymer activated by N-hydroxysuccinimide/N,N-dicyclohexylcarbodiimide (NHS/DCC) (Figure 3) (Mero and Campisi, 2014). Thanks to the excellent biocompatibility of HA, this polymer has also been used to bind to peptides. Besides, the broad range of HA MWs, and its abundant carboxyl groups facilitate its chemical binding to proteins or peptides (Zhang et al., 2021).

### Hyaluronic Acid Hydrogels

Hydrogels possess three-dimensional (3D) polymeric networks with good biocompatibility combined with low surface tension and high water content, providing hydrodynamic properties similar to those of natural organs, and their soft nature minimizes irritation to surrounding tissues. The above-mentioned properties underpin the wide application of hydrogels in biomedical research, such as bioreactors, tissue-engineering scaffolds, diagnostics, and drug delivery systems (Rao, 2020). HA is a promising building block to prepare a hydrogel matrix with a designed morphology for biomedical applications (Highley et al., 2016). To obtain HA-based hydrogels, chemical modification, gelling agents, or covalent cross-linking are often necessary. Different approaches have been proposed to design HA hydrogel matrices, such as enzymatic or disulfide cross-linking, click chemistry reactions, supramolecular assembly by inclusion complexation, etc. (Trombino et al., 2019). HA-based hydrogels are promising for drug and gene delivery, as well as stimulus-controlled drug delivery enabled by different triggers.

### Hyaluronic Acid-Based Nanoparticles

HA and other biomaterials can be prepared in multiple nanoformulations, such as nanocapsules, micelles, nanocomplexes, NPs, and nanogels (Tripodo et al., 2015). In particular, self-assembled structures (NPs, nanogels, and micelles) can be prepared by combining the hydrophilic skeleton of HA with other hydrophobic molecules, to optimize the solubility and stability of the encapsulated drug. Consequently, these self-assembled nanosystems are expected to control drug release and prolong the blood circulation (Cho, 2020).

### Hyaluronic Acid Micelles as Versatile Carriers

**FIGURE 4:**
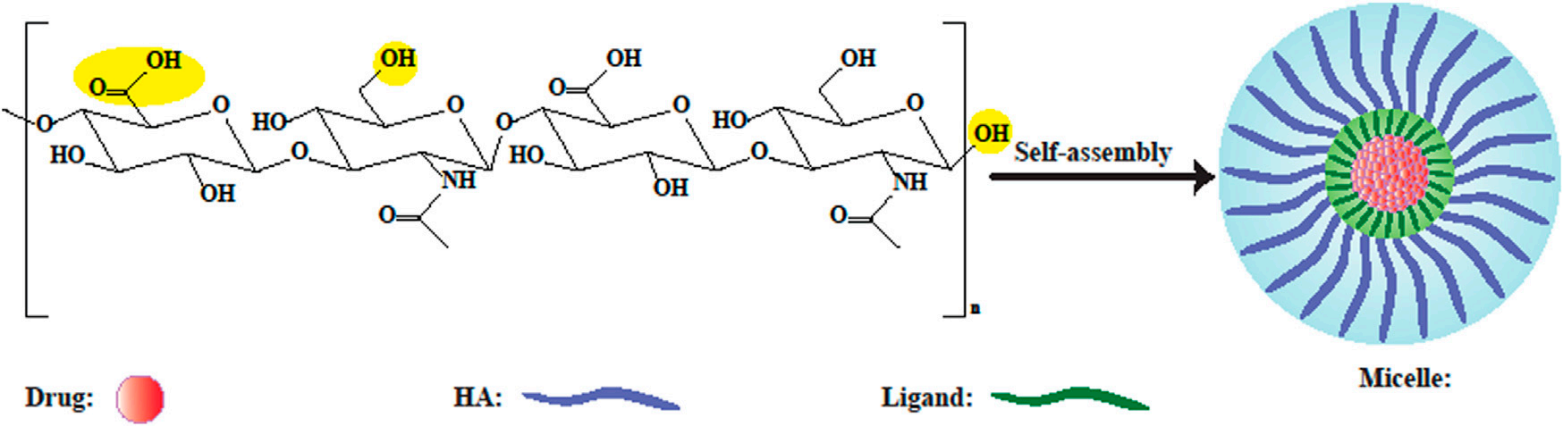
Schematic illustration of HA-based polymeric micelles. Yellow circles depict probable sites for modification. In aqueous solution, self-assembled micelles can be formed by conjugating amphiphilic HA-ligands to hydrophobic drugs, or by encapsulating drugs inside them (Kim et al., 2019).

Micelles can be formed by self-assembly of HA to create amphiphilic nanoconstructs. Micelles with 20–80 nm diameter form a colloidal suspension with an amphiphilic nature (Figure 4). Micelles with a smaller size could not deliver a sufficiently large amount of chemotherapeutic drugs to cancer tissue (Kim et al., 2018). Nevertheless, the bioavailability and half-life of the drug-loaded HA micelles can be increased due to the efficient targeted transportation of hydrophobic drugs to cancer site (Choi et al., 2012). An important problem with the intravenous injection of hydrophobic molecules, is their aggregation leading to embolization of blood capillaries, which can be alleviated by solubilizing hydrophobic drugs in appropriate carriers (Tuncer Degim and Celebi, 2007). Topical ocular drug delivery using polymeric micelles facilitates the penetration of drug into the eye tissue either through the corneal or conjunctival-scleral routes, due to the very small size of micelles. HA has been attracted interest as a micellar hydrophilic polymer because of its hydrophilicity, as well as covalent binding potential to hydrophobic drugs due to having both carboxyl and hydroxyl groups. Furthermore, additional advantages of HA in micellar formulations compared to other hydrophilic polymers, are the binding and uptake of micelles by recognizing specific receptors. Low MW HA (less than 10 kDa) is mostly used to form stable micelles, since higher sizes are not very suitable in this regard (Zhang et al., 2021).

### Hyaluronic Acid Nanogels

**FIGURE 5:**
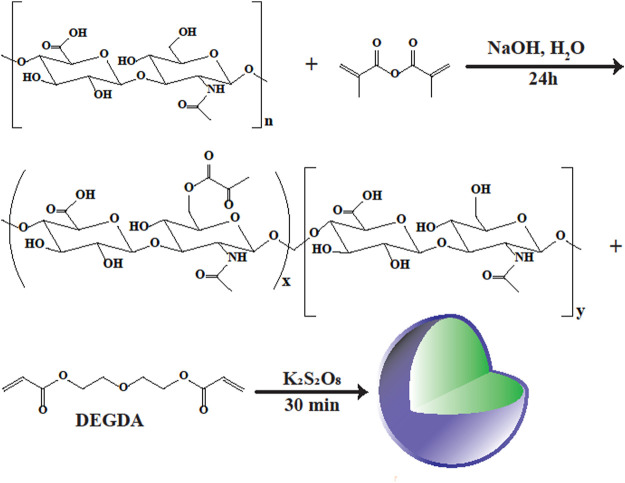
Synthesis route of HA nanogels. In order to present vinyl groups into HA, the sodium hyaluronic acid was modified with methacrylic anhydride, and the methacrylated hyaluronic acid (MAHA) was attained. Then, the HA nanogels were acquired by the copolymerization of MAHA with the ester bond containing cross-linker, di(ethylene glycol) diacrylate (DEGDA), in a aqueous solution (Yang et al., 2015).

Among the benefits of nanogels, is avoiding the rapid degradation of HA in the bloodstream. Various HA-based nanogels have been designed for drug delivery. For instance, HA nanogels were prepared by a cross-linking reaction between HA and divinylsulfone followed by polyaspartylhydrazide in water-in-oil (w/o) microemulsion using surfactants (Figure 5) (Yang et al., 2015). Also, HA nanogels could be prepared using modified HA either thiolated or in a hydrazide form in w/o emulsion-derived aqueous nanodroplets. Considering the high stability of nanogels in blood and the intrinsic bioactivity of HA, methacrylated HA (MAHA) was prepared by HA functionalization with vinyl groups, and then linked to DEGDA (diethylene glycol diacrylate), a hydrophilic cross-linking monomer, to form HA nanogels sensitive to enzymes in aqueous solution (Yang et al., 2015).

To combat bacterial infections, various antibiotics have been encapsulated in HA nanogels (Montanari et al., 2018; Mohammed et al., 2022). In this sense, Montanari et al. formulated HA nanogels encapsulated with levofloxacin (LVF) or gentamycin (GM) for targeting intracellular bacteria and infected human keratinocytes (Montanari et al., 2014). Recently, Liu et al. extended the performance of HA nanogels in antimicrobial delivery even further (Liu et al., 2021). They described a unique “on-demand” delivery of composite nanosystems based on the triple regulated release of inclusion composites (IC), polymeric NPs, and HA nanogels (Liu et al., 2021). The investigation’s ultimate goal was to efficiently remove *S. aureus*. Enrofloxacin was integrated into IC and then disseminated in poloxamer 188 coated nanogels created using ionic complexation of chitosan and HA. Nanosystems were created with mean EE percent, diameters, and PDI of 95.4 percent, 118.8 nm, and 0.26, respectively. By integrating IC into the HA nanogels, the nanosystem obtained multifunctional properties by releasing enrofloxacin at the infection site in a dual pH/HAaseresponsive manner, preventing premature drug release. Additionally, the composite nanosystems could be absorbed onto the surface of *S. aureus*, resulting in improved antibiotic activity (Liu et al., 2021).

## Hyaluronic Acid-Based Nanomaterials and Bacterial Infections

Despite initial success in controlling infections following the advent of antibiotics, emerging infectious diseases and the emergence of AMR have become one of the most serious international health challenges threatening humanity. Therefore, the discovery of innovative methods that reduce the toxicity of natural cells, increase antibacterial efficiency, and reduce the progression of bacterial resistance, is gaining popularity in research.

Nanomedicine, or the use of nanoscale materials to detect and cure diseases, provides a solution to this pressing problem (Soares et al., 2018). Although in comparison to other medical diseases like cancer and cardiovascular disease, the use of nanodrugs for the treatment of bacterial infections is still in the early stage, several types of nanodrug delivery systems, organic, and inorganic materials due to their increased antibacterial potential are reported in the literature (Misra et al., 2010; Wang et al., 2021).

Polymer-based NPs have received significant attention in antibacterial applications due to their biocompatibility, high drug loading capacity, ease of surface and chemical modification, and microenvironment responsiveness. In this context, silver nanoparticles (AgNPs) have recently become widely utilized for wound dressing and host tissue protection from external damage (Mohammed et al., 2022).

Usually, wound dressings provide better healing through an acceleration of the various stages of wound repair, and by covering the wound surface to prevent moisture loss. An effective wound dressing should meet the following criteria: 1) provide a moist environment; 2) prevent secondary infection of the wound; 3) absorb exudate and wound fluids; 4) disperse wound necrotic tissue; 5) maintain wound humidity; 6) induce secretion of growth factors; and 7) be non-immunogenic, biocompatible and elastic (Lin et al., 2001). Wound dressings consist of various components based on the wound types and healings mode. These materials can be synthetic polymers (poly-lactic acid, silicone rubber, polyurethane) or natural (chitosan, alginates, collagen, gelatin) (Dagalakis et al., 1980). In this respect, natural materials (green chemistry) are considered better alternatives to hazardous materials for human health and the environment. However, owning their low toxicity, biological activity, and ready availability AgNPs have long been recognized as a powerful inhibitor of microbial growth. Nevertheless, due to their toxicity, applications of AgNPs are limited, and one green synthesis strategy is the preparation of AgNPs using soluble starch as both the reducing agent and stabilizer molecule (Vigneshwaran et al., 2007). Another solution to this problem is to coat silver nanoparticles with various biologically active polymers to achieve biocompatible and environmentally friendly products (Montanari et al., 2018; Walvekar et al., 2019). Based, the natural molecules have been used as stabilizing agents in the AgNPs synthesis procedure along with other reducing agents (Raveendran et al., 2003; Abdel-Mohsen et al., 2012). As an interesting natural polymer, HA has generated increasing interest in the past 2 decades to develop HA-NPs as a targeted antibacterial nano drug delivery system (Sikkema et al., 2021). In this light, Abdel-Mohsen and others evaluated the antibacterial potential of HA fibers combined with AgNPs. To make a transparent solution, hyaluronic acid was dissolved in an aqueous solution of sodium hydroxide. This solution was then utilized to generate fibers using a wet-spinning approach (Abdel-Mohsen et al., 2013). Subsequently, the HA fibers were used as a capping agent (template) and stabilizing factor to produce AgNPs. The structural analysis and purity of HA-AgNPs were carried out *via* transmission electron microscopy (TEM), dynamic light scattering (DLS), scanning electron microscopy (SEM), energy-dispersive X-ray spectroscopy (EDX), ultraviolet-visible (UV/VIS) spectroscopy, nuclear magnetic resonance (NMR) spectroscopy, Fourier transform infrared spectroscopy (FTIR), thermal analysis, and X-ray photoelectron spectroscopy (XPS). The incorporation of AgNPs into HA fibers provided remarkable antibacterial activity against *S. aureus* and *E. coli* depending on the NP size. Of note, these formulations (10 and 40 nm diameter NPs) up to 100 μg/ml had no toxic effects on mouse fibroblast cells (NIH/3T3), making those promising candidates for antimicrobial wound dressings.

Diabetic foot ulcers are a serious complication of diabetic mellitus, which often develop as a consequence of peripheral neuropathy and ischemia (William and Keith, 2003). It is known that the healing of DFUs is a complicated process, and is often incomplete owing to heavy wound exudate and polymicrobial contamination. DFUs frequently lead to an increased risk of amputation (Jude et al., 2007). Clinical studies have shown that a high bacterial load in the wound reduces the probability of complete healing, and can also sometimes lead to sepsis and death (Nelson et al., 2006). Therefore, reducing the microbial load in the infected site could help to overcome the complications of DFUs. Another major obstacle in DFUs treatment is colonization (about 30%) with methicillin-resistant *S. aureus* (MRSA) isolates. On the other hand, the presence of antibiotic resistant bacteria in DFUs further increases the risk of amputation and death. Among various antibacterial agents, chitosan (CS) (a natural polymer composed of glucosamine and N-acetyl glucosamine) can efficiently heal DFUs due to its beneficial properties (biodegradability, biocompatibility, and non-toxicity) (Jayakumar et al., 2011; Paul and Sharma, 2012). Recently, CS-based antibacterial NPs have been investigated as wound dressings (Archana et al., 2013).

All of the research described in this part validate the potential use of HA as a promising polymer for the formulation of HA-NPs for the prevention and eradication of bacterial infections. These NPs can improve antibacterial agent localization by delivering smart targeted antibacterial therapy, decreasing the side effects of loaded drugs, and regulating the release of loaded antibacterial agents (Zhang et al., 2020; Ferreres et al., 2021).

Polyurethane (PU) is another well-recognized component of wound dressings with good biocompatibility and mechanical properties (Dos Santos et al., 2018; Bužarovska et al., 2019). Many efforts have been made to use natural polymers to improve the efficiency of synthetic polymers such as PU (Unnithan et al., 2012). Although antibacterial activity is a beneficial property of wound dressings, this property is absent in PU, resulting in a delay in the wound healing process. Other problems caused by wound infections are systemic infection, bacteremia, or even death (Li et al., 2019; Qu et al., 2019). Therefore, wound dressings impregnated with antibacterial materials have been proposed to diminish the risk of infection and bacterial colonization in the wound site (Liu et al., 2019). In addition to other biological effects (anti-inflammatory, antiviral, antifungal), propolis has been applied for a long time as a practical antibacterial substance and a potential facilitator of wound healing (Roy et al., 2010; Khoshnevisan et al., 2019). This resinous and adhesive natural complex is formed by the salivary secretion of bees, and plant exudates, and is found in beeswax. The antibacterial properties of propolis are attributed to several components, including aldehydes, phenolic acids and esters, steroids, flavonoids, amino acids, and ketones (Khoshnevisan et al., 2019; Sharaf and El-Naggar, 2019). Studies have shed some light on the beneficial effects of propolis as a wound dressing platform (Khodabakhshi et al., 2019).

Eskandarinia et al. synthesized a nanofibrous wound dressing using an ethanolic extract of propolis (EEP) combined with PU-HA (Eskandarinia et al., 2020). Subsequently, the nanofibrous scaffolds were characterized by FTIR, thermal analysis, morphological and microstructural investigations, mechanical properties, antibacterial activity, water absorption measurement, and *in-vivo* and *in-vitro* assays. When compared with other PU-HA EEP fibers, the PU-HA/1% EEP and the PU-HA/2% EEP displayed a larger inhibition zone against *S. aureus* (2.36 ± 0.33 and 5.63 ± 0.87 mm respectively), *E. coli* (1.94 ± 0.12 and 3.18 ± 0.63 mm respectively). The PU-HA/1% EEP dressing showed no cytotoxicity on L929 fibroblast cells, and better biocompatibility compared to PU-HA/2% EEP. Likewise, the PU-HA/1% EEP fibers accelerated healing and wound closure in excisional wounds in rats. Histopathological results showed that the PU-HA/1% EEP formulation facilitated collagen accumulation and dermal organization at the repaired wound site, in comparison with other treatments. These results showed that EEP (1 wt%) incorporated into the PU-HA nanofibrous composite could be used as a practical and biocompatible wound healing dressing with additional antibacterial activity.

Since some antibiotics which are prescribed as conventional treatments for human pathogenic infections can be cytotoxic, expensive, and can cause drug resistance (O’Shea and Moser, 2008), the need for novel alternative antimicrobial materials against a wide range of pathogens has gained great attention (Zasloff, 2002; Hajipour et al., 2012). A naturally occurring polyphenolic pigment called curcumin (Cur) can be isolated from turmeric (*Curcuma longa*) and has been shown to exert potential microbicidal activity against a variety of pathogens (Schraufstätter and Bernt, 1949; Aggarwal et al., 2003). In addition to antimicrobial and anticancer effects, Cur itself can be used as a reducing agent to prepare stable AgNPs or AuNPs (Sreelakshmi et al., 2013; Loo et al., 2016). Furthermore, it was reported that the combination of Cur with AuNPs or AgNPs could provide antibacterial synergism against pathogenic microorganisms. To overcome the cytotoxicity of metal NPs and provide sustained release in the infected site, various polymeric matrices have been investigated as delivery vehicles (Archana et al., 2015).

Madhusudana Rao et al., prepared sodium HA stabilized Cur-Ag hybrid NPs under constant stirring conditions, and characterized the samples by XRD, FTIR, and UV-VIS spectroscopy (Kummara et al., 2017). Structurally, the Cur-Ag hybrid NPs presented a spherical profile with 5–12 nm size, which was directly affected by the Ag + ion concentration as measured by TEM. In the next step, the hybrid NPs were applied to coat a CS-impregnated fibrous cellulose filter paper using polyelectrolyte complex formation, and the uniform distribution was confirmed by morphological examination. The findings confirmed the potential of HA as a stabilizing agent of Cur and Cur-Ag NPs mediated by hydrogen bonding interactions. The results of disc diffusion assays showed good synergistic antibacterial effects of the Cur-Ag NPs in the biocomposite matrix against *E. coli*. This biopolymer-based hybrid system had a green nature and synergistic antibacterial activity, highlighting its potential as a wound dressing biocomposite.

Graphene oxide (GO) nanosheets (NS) are a graphene derivative with great promise as an antibacterial material against various bacteria, with good biocompatibility and low toxicity toward mammalian cells (Ji et al., 2016; Xia et al., 2019). The mechanism underlying the antimicrobial activity of GO NSs was proposed to involve the removal of phospholipids from bacterial membranes caused by the formation of reactive oxygen species (ROS), followed by impairment of cell membrane integrity and bacterial death (Tu et al., 2013; Xia et al., 2019). The exact mechanism of action and the selective antibacterial activity of GO nanosheets without unwanted harmful effects on normal mammalian cells is still unclear (Tu et al., 2013). Multicomponent nanocomposites based on GO (nanometals, polymeric quaternary ammonium salts, and antibiotics) have been developed to solve the problems with the individual components (Wang et al., 2015). Of interest, the conjugation of CS to GO (GO-CS nanohybrids) improves the antimicrobial and biosafety profiles and prolongs the biological half-life of CS for extended coverage of wound dressing composites at the injured site (Joz Majidi et al., 2019).

Copper (Cu) is a cost-effective antibacterial metal that efficiently eliminates microbes through the release of Cu ions (Cu2+) from the surface, resulting in respiratory chain collapse and impaired DNA replication in bacterial cells (Li et al., 2016a). Moreover, the redox reactions of CuNPs can oxidize lipids and proteins resulting in microbial death (Wei et al., 2010). Cu2+ has also been proposed to exert pro-angiogenesis effects by stimulating vascular endothelial growth factor (VEGF) and hypoxia-inducible factor (HIF-1α) (Kohara et al., 2013). Therefore, Cu-embedded wound dressings could be used to manage bacterially infected wounds due to the angiogenic and anti-microbial effects (Li et al., 2016b). Meanwhile, CuNP-conjugated GO membranes papered showed increased antimicrobial activity through an *in situ* chemical reduction process (Xu et al., 2020).

Ying Yang et al., manufactured chitosan/hyaluronic acid (CS/HA) dressings incorporating GO/Cu (CS/HA/GO/Cu) using sodium trimetaphosphate (STMP) cross-linking and vacuum freeze-drying (Yang et al., 2020). The GO-decorated C/H/GO dressings and C/H dressings were employed as controls. They found that an acidic pH value accelerated the release of copper (Cu2+ and CuNPs) from the dressing scaffolds. The best *in vitro* antimicrobial performance was found with CS/HA/GO/Cu dressings, against two *S. aureus* strains (ATCC25923 and ATCC35984). All three dressings showed acceptable cytocompatibility in murine fibroblasts (NIH/3T3-L1). CS/HA/GO/Cu synergistically improved wound healing. Histopathology showed a decreased infiltration of inflammatory cells, enhanced angiogenesis, and improved cutaneous organization in infected skin wounds. There was no sign of damage to the tissue structures of the heart, liver, lungs, or kidneys in any of the four groups. They suggested that multifunctional CS/HA/GO/Cu dressings could be an ideal alternative wound dressing to treat and prevent infected wounds in plastic surgery clinics.

Titanium and its various alloys have been considered beneficial biomaterials in implant dentistry, owing to their good biocompatibility and outstanding physicochemical properties (Neoh et al., 2012). Nevertheless, some dental implant procedures can result in implant-associated infections (IAIs), leading to dental implant failure, psychological trauma, and high social and financial costs (Darouiche, 2004). Up to 65% of hospital-acquired infections have been attributed to biofilm formation, with a profound effect on providers of many therapeutic procedures (Mah and O'Toole, 2001). The management of peri-implantitis, as well as peri-implant mucositis,has become a major clinical problem, due to the need for long-term antibiotic treatment and repeated surgical procedures (Camargo and Van Sickels, 2015). Biofilms are generated by the attachment and aggregation of bacteria on the implant surface and have been cited as a prime cause of infection in the mucosa and bone located near an implant site (Koseki et al., 2014). To form biofilms, unicellular microorganisms must co-aggregate and develop an organized community embedded within an exopolysaccharide matrix. Biofilm increases drug resistance and bacterial tolerance to many antibacterial therapies (Esposito et al., 1998), emphasizing the need to discover long-lasting antibacterial coatings applied to the surface of titanium implants to prevent biofilm formation and thus reduce the incidence of IAI.

To facilitate the deposition of antibacterial coatings, the implant surfaces can be pretreated by physicochemical techniques. The modification of the physical surface can be carried out by ion beam implantation, physical vapor deposition, or lithography-based approaches (Yeo, 2014). Chemical reactions are the most efficient modification approach, which includes peroxidation, acid etching, anodic oxidation, alkali treatment, attachment of functional molecules by covalent cross-linking, hydrothermal modification, and chemical vapor deposition (Zhong et al., 2016). Since the application of the above-mentioned methods is complex, involving steps of priming, hazardous chemical reagents, and mass production procedures, an improved method for the immobilization of antibacterial coatings on the Ti surfaces would be highly desirable. In this context, a new “phase-transited lysozyme” (PTL) was used to provide an active surface in Ti with some advantages (e.g., simple, fast, and low-cost preparation along with green technology) compared to some traditional surface functionalization procedures. The PTL product can robustly adhere to the substrate, because it leads to the development of an amyloid-like microfiber net due to the β-sheet transition of lysozyme microfibers.

Xue Zhong et al., developed a novel PTL-based priming layer on the Ti surface by dipping Ti discs into a tris (2-carboxyethyl) phosphine (TCEP) and lysozyme hybrid mixture to functionalize the Ti surface (Zhong et al., 2016). Then, a layer-by-layer (LbL) self-assembly approach produced multilayered coatings of AgNP-anchored HA and CS on the surface of PTL-pretreated Ti substrates. The data from XPS and SEM analysis confirmed a necklace-like PTL structure with 0.5–1 μm diameter fibers, and successful loading of the self-assembled multilayer onto the Ti substrate. The antibacterial efficacy of AgNP-loaded multilayer coatings was 100% after the first 4 days followed by a relatively sharp reduction (65%–90%) in the next 14 days. The controlled Ag release over 14 days provided the prolonged antimicrobial activity of the implant coatings until the mucosa could heal. Also, controlling the release rate and concentration of Ag decreased any possible cytotoxicity of the AgNPs. Therefore, the PTL priming method could allow the fabrication of prolonged antibacterial multilayer coatings on Ti surfaces through an LbL self-assembly method. This approach could inhibit IAIs and accelerate osseointegration in the first step of implantation.

Antimicrobial peptides (AMP) are compounds that have been identified as potentially effective therapeutic candidates for the treatment of bacterial infections. These components, however, demonstrated limited biostability and bioavailability, as well as severe toxicity (Teixeira et al., 2020). To combat these challenges, hyaluronic acid-based nanogels have recently been investigated as viable nanoplatforms for antimicrobial peptide and peptidomimetics delivery. In this regard, HA nanogels were prepared by cross-linking HA with a thiolated alkyl chain and loaded with LLKKK18 peptide to treat pulmonary mycobacterial infections (Silva et al., 2016). The results showed that loaded nanogels had no effect on bone marrow-derived macrophages at concentrations up to 100 M, which is more than 20-time higher than the lethal concentration of free peptide. Moreover, HA nanogels can bind to the overexpressed CD44 receptor on the surface of activated macrophages, resulting in selective targeting of LLKKK18 to mycobacteria. *In vitro* treatment of macrophages with LLKKK18-loaded nanogels lowered intracellular levels of *Mycobacterium tuberculosis* and *Mycobacterium avium*. *In vivo* tests with LLKKK18-loaded nanogels revealed a significant decrease in infection levels in mice infected with *M. tuberculosis* or *M. avium* (Silva et al., 2016).

Bacterial biofilms are one of the major global health concerns because they have the potential to tolerate host defense systems and antibiotics, therefore, they contributed to antibiotic resistance as well as chronic and recurrent infections (Sharma et al., 2019). Recently, Fasiku et al. prepared a nanogel *via* crosslinking HA solution with divinyl sulfone for the co-delivery of NO and AMP against bacteria and biofilms (Fasiku et al., 2022). *In vitro* antibacterial tests revealed that the NO-AMP-loaded nanogel demonstrated a greater antibacterial/antibiofilm activity when compared to NO alone. The antibiofilm results showed that catheters exposed to nanogel loaded with AMP/NO reduced biofilms of MRSA and *P. aeruginosa* by 12.5 and 24-folds, respectively, as compared to only NO, while nanogel loaded with only NO reduced biofilms of MRSA and *P. aeruginosa* by 7 and 9.4-folds, respectively (Fasiku et al., 2022).

Synthetic anti-biofilm peptides that act against biofilms are progressively reported in the literature (De La Fuente-Núñez et al., 2016; Dostert et al., 2019). Anti-biofilm peptides are a subclass of antimicrobial peptides that provide prospective broad-spectrum therapeutics for the treatment of bacterial biofilms, albeit some exhibit host toxicity *in vivo*. In one study, nanogels (174–194 nm) made of modified hyaluronic acid were used to encapsulate the anti-biofilm peptide DJK-5 *in vivo* (Kłodzińska et al., 2019). According to the findings, the dose of DJK-5 that could be supplied intravenously to mice without causing toxicity was more than doubled after encapsulating in nanogels, Subcutaneous dosing reduced the toxicity of DJK-5 in nanogels fourfold compared to non-formulated peptide, without impairing DJK-5’s anti-abscess efficacy (Kłodzińska et al., 2019). These findings support the use of HA nanogels to increase the compatibility and safety of antimicrobial peptides, anti-biofilm peptides, and peptidomimetics delivery due to their capacity to improve antibiofilm action, bioavailability, antibiotic localization on-site, and eradicate the cytotoxicity of loaded drugs.

While significant efforts have been made to demonstrate the enhanced potential of HA-NPs for bacterial eradication and detection, these nanosystems are still in their infancy, and countless additional studies are required to acquire regulatory approval. The manufacturing, synthetic modification, accurate characterization, and optimization of HA-NPs, as well as their biosafety and *in vivo* pharmacokinetics, should be done precisely by clinical requirements. Furthermore, greater efforts are needed to address the challenges involved with scaling up these formulations into cost-effective solutions. Additional research is needed to completely understand the interactions between these nanocarriers and the targeted biomolecules, such as bioinformatics methods and *in vitro* binding affinity calculations. *In silico* investigations can aid in determining the stability of produced HA-NPs by simulating experimental settings with virtual ones.

Supplementary Table S1 lists some studies on the application of HA-based nanoparticles in the treatment or prevention of bacterial infections.

## Conclusion

The bactericidal activity of HA is not particularly well-known and is thought to be dependent on its concentration, molecular weight, bacterial species, and the type of interaction with bacterial cells. However, the incorporation of HA into antimicrobial materials can provide direct contact between the pathogenic bacteria and the dressing composites. Therefore, the combination of HA with other antimicrobial materials, such as silver or polyhexanide polymers, provides efficient antimicrobial activity against bacterial strains, especially for the healing of hard-to-treat infected wounds. This approach opens a new window in tissue engineering as well as wound healing. This approach may allow the controlled delivery of an additional suitable concentration of an antimicrobial compound during the whole time of treatment. Moreover, HA-based antimicrobial coatings may be applied to medical devices and surgical implants to reduce the risk of developing hospital-acquired infections, which are often antibiotic-resistant.
