# Supplementary material for: Hyaluronic Acid-Based Nanomaterials as a New Approach to the Treatment and Prevention of Bacterial Infections
Source: Front Bioeng Biotechnol. 2022 Jun 8;10:913912. doi: 10.3389/fbioe.2022.913912 (PMC9213665; doi:10.3389/fbioe.2022.913912)
Supplement: Supplementary file 1 [file Table1.docx]

**Table S1.** Hyaluronic acid-based nanomaterials for antibacterial applications

| **HA-based nanomaterial** | **Target microorganisms** | **Model *(in vitro/ in vivo/* Huma*n)*** | **Mechanism of antibacterial activity** | **Results** | **Ref** |
| --- | --- | --- | --- | --- | --- |
| HA-Ag NPs | *S. aureus*  *E. coli* | *In vitro* | Interfered with cell wall formation, prevented protein expression, interfered with nucleic acid formation, inhibited metabolic pathways | Wound healing dressing | (87) |
| Chitosan–HA/nAg | *E. coli,* methicillin-resistant *S. aureus (MRSA) S. aureus, P. aeruginosa, K. pneumoniae* | *In vitro* | Bound to bacterial cell membranes, membrane damage via interactions with sulfur-bearing proteins, penetrated into bacteria, impaired DNA synthesis, destruction of respiratory chains, prevented cell division, bacterial cell death by free radical formation and oxidative stress induction | Nanocomposite sponges can be used as wound dressing for antibiotic resistant bacterial-infected DFUs | (142) |
| PU-HA/2% EEP | *S. aureus*  *E. coli* | *In vitro*  *In vivo* | Antimicrobial activity of propolis based on flavonoids and phenolic compounds as potent antibacterial agents; caffeic acid in EEP exerted antibacterial activity; release of EEP from wound dressing allowed penetration of flavonoids, aromatic acids and esters to the wound site | Good biocompatibility, promoted wound healing, antibacterial activity for biomedical purposes | (106) |
| OHA/HA-ADH/O-HACC  OHA/HA-ADH/N-HACC | *S. aureus*  *E. coli*  *P. aeruginosa* | *In vitro* |  | OHA/HAADH/N-HACC and OHA/HA-ADH/O-HACC formed antimicrobial hydrogel wound dressings for infected open wounds | **(15)** |
| GO-HA-AgNPs. | *S. aureus* | *In vivo*  In vitro | Binding of AgNPs to bacterial cells induced the formation of  reactive oxygen species (ROS);  GO can damage cell membranes | Novel antimicrobial compounds for synergistic antibacterial activity with no mammalian cell damage | (143) |
| HA stabilized CUR-AgNP | *E. coli* | *In vivo* | AgNPs bound to bacterial cell membranes, internalized by bacterial cells, damaged the respiratory chain by releasing Ag+ ions, caused cell death; AgNPs and Cur had synergistic effect against bacteria, prevented ATP synthesis | Antibacterial paper dressings were effective for infected wound healing | (115) |
| ALG/HA/usSN | *MRSA,*  *multidrug resistant S. epidermidis (MRSE),*  *P. aeruginosa, E. coli, A. baumannii,*  *K. pneumoniae* | *In vitro* | Ag+ ions directly affected bacterial cell membranes, inhibited respiratory chains, inhibited membrane formation, stopped ATP synthesis | Developed easy to prepare multifunctional hydrogels, could be used in clinical settings | (144) |
| CS-G/HA-NPs | *S. aureus*  *E. coli*  *B. subtilis* | *In vitro*  *In vivo* | Chitosan disrupted bacterial cells,  increased bacterial membrane permeability | CS-G/HA-NPs hydrogels as adjustable drug delivery vehicles, promotes wound healing | (145) |
| OSA-HA-DJK-5 | *P. aeruginosa* | *In vivo* | Bound to the surface of bacteria, damaged membranes by forming pores | Increased the safety of anti-biofilm and antimicrobial peptides, administered by intravenous and subcutaneous routes | (141) |
| HA/CEL/GUMBOS | *S. aureus ATCC*  *29213* | *In vitro* |  | Possible use as patches for wound healing | (146) |
| C/H/GO/Cu | *MRSA (ATCC35984 and ATCC25923* | *In vitro*  *In vivo* | Inhibited bacterial attachment and biofilm formation, bacterial cell death, interplay of GO damaged integrity of cell membranes, inhibited protein, lipid and DNA/RNA synthesis, bacterial growth was inhibited by Cu ions by increasing membrane permeability, inhibited respiratory chains, ROS-mediated damage of DNA/RNA, bacterial cell membranes were irreversibly damaged when cultured on C/H/GO/Cu dressing scaffolds | Wound dressings to treat intractable bacterial-infected wounds | **(126)** |
| collagen I/hyaluronic  acid/quaternized chitosan multilayer modified titanium coatings (Col I/HA/HACC multilayer modified TCs) | *S. aureus (ATCC 25923)*  *MSRA ATCC 43300)*  *clinical isolate*  *MRSE 287* | *In vitro*  *In vivo* |  | Prevented implant associated infections | (147) |
| Ti-PTL-HA-CS/Ag | *S. aureus, ATCC 25923* | *In vitro* | Ag^+^ release from AgNPs inhibited DNA replication of bacteria,  killed bacteria by triggering the formation of ROS, increased bacterial membrane permeability | AgNP coatings on medical equipment, such as bone cement, wound dressings and catheters | (134) |
